# Socio-economic and cultural factors that influence the labor insertion of University Graduates, Peru

**DOI:** 10.1016/j.heliyon.2021.e07420

**Published:** 2021-06-25

**Authors:** Pedro Jesús Maquera-Luque, José Luis Morales-Rocha, Cynthia Milagros Apaza-Panca

**Affiliations:** a Universidad Nacional de Moquegua, Moquegua, Perú; b Facultad de Administración Hotelera y de Turismo, Universidad Nacional de Frontera, Sullana, Piura, Perú

**Keywords:** Social factors, Economic factors, Cultural factors, Labor insertion, Multivariate model, Logistic regression

## Abstract

Access to the labor market by graduates of the National University of Moquegua is limited by a wide range of socioeconomic and cultural factors. The study aimed to develop a multivariate model to identify the socioeconomic and cultural factors that influence labor insertion of graduates of the National University of Moquegua, 2019. The type of research according to its purpose was basic and the non-experimental cross-sectional design, with a stratified random sample with proportional allocation with a significance level of 5% and a sampling error of 7%. The data collection technique was the survey and two validated and reliable instruments were applied. The population consisted of 537 graduates, with a sample of 121 graduates from six Professional Schools. The results of the application of logistic regression models indicate that the employment status (Wald = 21.179 and p-value = 0.000), basic electricity services (Wald = 4.567 and p-value = 0.033), the preference for movies (Wald = 6,136 and p-value = 0.013), and the communications media: TV and radio (Wald = 4.962 and p-value = 0.026) significantly influence the labor insertion of graduates of UNAM. It is concluded that both the working condition, electricity services, the preference for movies and communication media like TV and radio significantly influence the labor insertion of graduates of the National University of Moquegua.

## 1 Introduction

Universities are institutions responsible for the training of a part of the active population, being obliged to continually review their offers, adapting them with greater precision and objectivity to the current market demands and requirements of what can be considered as professional demands from the future (Rodríguez Sabiote, 2007). Currently, universities must assume responsibility in the insertion of graduates in the labor market to improve their offer in education and training (Loncomilla et al., 2012). For the National Association of Universities and Institutions of Higher Education - ANUIES, relevance is one of the central criteria that have guided the design of educational policies at the higher level in recent years, an issue that is evidenced in the coherence that exists between the objectives and the graduate profiles of the study plans, in addition to the work market; the latter being a challenge that countries face to improve the competitiveness of the sector (Brito Laredo et al., 2017).

Today's universities are increasingly becoming sites for the production of new knowledge in accordance with collective needs, causing the higher education system to have relevance in the new processes of productivity and competitiveness, and the institutions that are part of the aforementioned system have been required to address institutional evaluation as one of the most acceptable mechanisms for improving university excellence, establishing criteria to evaluate the inputs, processes and products that they generate (Escobedo Portillo et al., 2013).

Undoubtedly, García-Blanco & Cárdenas-Sempértegui (2018) the university has undergone a true revolution by incorporating training for labor insertion among its objectives. Although Higher Education has always been aimed at training professionals, due to its objective are focused on certifying qualified professionals, knowledge acquired. Hence, it was noted that there was little interest in following their graduates and therefore, their capacity to incorporate themselves in the world of employment.

At national level, University Law No. 30220, in its Article 28 subsection 7, indicates that one of the basic conditions established by SUNEDU (Superintendencia Nacional de Educación Superior Universitaria – SUNEDU / National Superintendence of Higher University Education) for licensing refers to the existence of mediation and job placement mechanisms, that is, a job board or others. Nevertheless, National University of Moquegua (UNAM) has only about 6.3% certified professional out of 537 graduates. In other words, there is only 34 certified professionals from the all six professional schools.

Likewise, UNAM has a Directorate for Mediation and Labor Insertion, which is a university instance responsible for providing information regarding the employability and labor progression of UNAM graduates, constituting a bridge between the labor insertion needs of graduates and the requirements of the labor market, monitoring work trajectories and indicators of income and employability of those graduates holding a bachelor's degree, graduates holding a degree certificate and graduated ones. Despite the efforts made by the State and private sector to generate new sources of employment, there are still unemployed or underemployed people.

In this context, both people who offer their labor force and whom ask it for, they make up labor market. So, in this market context, there may be other factors in addition to the traditional economic ones, which explains the demand-supply imbalances; Other factors such institutional, social, cultural and demographic ones could help interpret in a deeper way those imbalances which refer (Muñoz, 2009; Valle Rivadeneyra, 2012). However, socioeconomic and cultural factors that influence the labor insertion of graduates of the UNAM are unknown, which leads us to wonder. In what way will a multivariate model make it possible to identify the socio-economic and cultural factors that influence the labor insertion of graduates of the National University of Moquegua?

Although job placement is related to people's lives and their aspiration to get a job, it should always be understood as a process, transition and established trajectories in each job market, with a clear impact on personal development (García-Blanco and Cárdenas-Sempértegui, 2018). Therefore, job insertion becomes a highly valued indicator, both in graduate studies, and in the accreditations to which Higher Education Institutions submit (Instituciones de Educación Superior - IES) (Zalapa Lúa et al., 2019). In the same direction, employability is given by the competences that would allow access to a job, to a new one in search of a better professional situation, to keep it, being related to the ability to perform functions and tasks in specific contexts (García-Blanco and Cárdenas-Sempértegui, 2018).

Thus, job placement is more linked to demand, employability is related to the competencies of each individual to access that job and respond adequately to what is required. The employability of a graduate is understood as the set of attributes (knowledge, abilities, skills, attitudes, values) that employers seek and that graduates must develop effectively in their professional schools, there is a great deal of mix personal qualities and beliefs to be found, the same ones that lie on employability skills that allow to become strong ambassadors for the organization (Patache, 2016; Vega et al., 2012). This being so, understanding socioeconomic processes provides the basis for inferring individual socioeconomic status (Gao et al., 2019).

Based what has been stated in paragraphs above, Higher Education is called to respond to the demands of the world of work, which means that universities must be informed of the expectations and demands of society, in order to anticipate their demands, preparing professionals who this needs at all times and in each sector. Therefore, the study aims to develop a multivariate model to identify the socioeconomic and cultural factors that influence the labor insertion of graduates of the National University of Moquegua, 2019.

## 2 Materials and methods

The type of research for the study according to its purpose was applied (Carrasco, 2009), and the non-experimental cross-sectional design. The technique used was the survey, and the instrument, the questionnaire. For the study, two instruments were used that were validated by expert judgment and their reliability was determined using Cronbach's Alpha. The variables were: socioeconomic and cultural factors (independent variable), and job placement (dependent variable).

The population was made up of 537 graduates of UNAM, information obtained from the Directorate of Academic Activities and Services (hereinafter DASA – Dirección de Actividades y Servicios Académicos), as shown in Table 1. The sample consisted of 121 graduates from the six UNAM Professional Schools, where stratified random sampling with proportional allocation was used, considering a significance level of 5% and a sampling error of 7%. Likewise, to select the graduates who were part of the sample, age was used as criteria, being those over 18 years old, graduated from the 6 professional schools and that their year of graduation corresponds to the first class, that is, from the year 2012 onwards. In addition, the sampling strategy is based on Seoane et al. (2007) and Martinez (2012) pointing out that in stratified random sampling, the population to be investigated is divided into relatively homogeneous subsets in relation to the study feature.

**Table 1:** Population of graduates of the National University of Moquegua, 2017

Professional Schools	Population
Public Management and Social Development	136
Agro-industrial Engineering	98
Mining Engineering	98
Systems Engineering and Informatics	71
Environmental engineering	90
Fisheries Engineering	44
Total	537

Stratified random sampling formula

Eq. (A.1) $$\begin{array}{l} n_{0}=\frac{1}{V}\sum W_{h}P_{h}Q_{h}\\ V={\left(\frac{E}{Z}\right)}^{2}\\ n=\frac{n_{0}}{1+\frac{n_{0}}{N}} \end{array}$$

Where:

*W*_*h*_: Proportion of elements of each professional school
*P*_*h*_: Proportion of female graduates from each professional school
*Q*_*h*_ = 1−*P*_*h*_
*E* = 5%: Sampling error
*Z* = 5%: Significance Level $Z_{0.05/2}=Z_{0.025}=1.96$ (statistical table value)

Replacing values:

$$V={\left(\frac{0.05}{1.96}\right)}^{2}=0.00065$$

$$n_{0}=\frac{1}{0.00065}\left(0.25\times 0.29\times 0.71+0.18\times 0.42\times 0.58+0.18\times 0.87\times 0.13+0.13\times 0.75\times 0.25+0.17\times 0.38\times 0.62+0.08\times 0.34\times 0.66\right)$$

$$n_{0}=156.8$$

$$n=\frac{156.8}{1+\frac{156.8}{533}}=121.4$$

n=121

The sample size with proportional allocation is shown in Table 2:

**Table 2:** Sample of graduates of the National University of Moquegua, 2017

Professional Schools	Population
Public Management and Social Development	31
Agro-industrial Engineering	22
Mining Engineering	22
Systems Engineering and Informatics	16
Environmental engineering	20
Fisheries Engineering	10
**Total**	**121**

### 2.1 Validity

The instruments were reviewed and validated by means of expert judgment, as shown in Table 3, from which we deduce that both instruments are valid, given that they have an average valuation of 90% for the labor insertion variable and 85% for the variables socioeconomic and cultural factors.

**Table 3:** Level of validity of the questionnaires according to expert judgment.

Experts	Labor Insertion	Socioeconomic and cultural factors
	%	%
Expert 1	90	80
Expert 2	85	85
Expert 3	95	90
Average rating	90	85

It should be noted that social factors considered for the study were: age, sex, specialty, marital status, number of children, graduate, type of housing, work activity, social organization to which the participant belongs, activities in their free time; economic factors such as: work status, average monthly economic income, service time, main material of housing, ownership of housing, basic services; and, cultural factors: language skills, TV preferences, media information, parents' degree of education, place of birth, place of residence, social networks.

### 2.2 Reliability

Castillo-Sierra et al. (2018) consider that, in the measure of reliability by means of Cronbach's Alpha coefficient, it is assumed that the items measured in Likert type scale measure the same construct and are highly correlated. So, for this reason the instrument's reliability criterion was determined by Cronbach's alpha coefficient of internal consistency, which requires a single administration of the measurement instrument and produces values that oscillate between one and zero, according to the values in Table 4.

**Table 4:** Values of reliability levels.

Values	Reliability levels
0.53 - less	Null reliability
0.54–0.59	Low reliability
0.60–0.65	Reliable
0.66–0.71	Very reliable
0.72–0.99	Excellent reliability
1.00	Perfect reliability

Table 5 shows a Cronbach's alpha coefficient value of α = 0.903, from which we deduce that the instrument used was of excellent reliability for the labor insertion questionnaire; and in the application of the questionnaire of socioeconomic and cultural factors, the Cronbach's alpha coefficient of α = 0.701 was obtained; from which it can be concluded that the instrument was very reliable.

**Table 5:** Labor insertion reliability statistics.

Instrument	Cronbach's alpha
Labor insertion questionnaire	0.903
Socio-economic and cultural factors questionnaire	0.701

## 3 Results

Next, the results obtained with respect to the socioeconomic and cultural factors that influence the labor insertion of graduates of the National University of Moquegua are presented.

In Table 6, it is observed that 41.3% of the graduates looked for a job through the media such as advertisements in the newspaper, the internet, etc., 15.7% through employment agencies, 34.7 % through direct contact with employers, 16.5% continued in the same company or institution where they carried out their pre-professional internships, 43% of graduates looked for a job through family and friends, 2.5% of graduates started their own business and 0.8% of graduates did not look for a job.

**Table 6:** What means did you use to look for a job?

Used means	Count	Percentage
Through the media (newspaper ads, internet, etc.)	50	41.3%
Through employment agencies/temporary work agencies	19	15.7%
Contacting the employer directly	42	34.7%
Continue in the same company or institution where I did my internship	20	16.5%
Through personal contacts (family, friends)	52	43.0%
None, I set up my own business	3	2.5%
None, I did not look for a job	1	0.8%

Table 7 shows that 44.6% of UNAM graduates consider that there is a job, but affirm that university studies are not enough to get a good job, 30.6% consider that there is not much offer of jobs in their specialty, 19% affirm that there is job opportunities but that the contracts are very precarious or do not ensure stability and 5.8% consider that there is a large job offer.

**Table 7:** How do you perceive the labor insertion from your university studies?

Perception	Frequency	Percentage
There is work, but university studies are not enough to get a good job	54	44.6
There is work, but the contracts are very precarious	23	19.0
There is a lot of job offer in my profession	7	5.8
There is not much job offer in my profession	37	30.6
Total	121	100

In Table 8, it is observed that 27.3% of the graduates of the National University of Moquegua were working in positions related to their specialty, 24.8% in positions that were not related to their specialty, 25.6 % were unemployed, but were previously working in positions related to their specialty, 15.7% of graduates were unemployed, but had worked in positions not related to their specialty, and 6.6% had not worked.

**Table 8:** What is your current employment situation?

Employment Situation	Frequency	Percentage
I work in a position related to my studies	33	27.3
I work in a position NOT related to my studies	30	24.8
Unemployed, having previously worked in a position related to my studies	31	25.6
Unemployed, having previously worked in a position NOT related to my studies	19	15.7
Unemployed, I haven't worked so far	8	6.6
Total	121	100

For the logistic regression analysis, classification Table 9 indicates that there is an 89.3% probability of success in the result of the dependent variable, when the independent variable is known.

**Table 9:** Classification tables between the observed and predicted values of the socioeconomic and cultural factors that influence the labor insertion of graduates of the National University of Moquegua.

Observed		Predicted		
		Employment Status		Correct percentage
		Does not work	Work	
Employment status	Does not work	47	11	81
	Work	2	61	96.8
Overall percentage				89.3

### 3.1 Socioeconomic and cultural factors that influence on graduates' labor insertion from National University of Moquegua

Social factors considered for the study were: age, sex, specialty, marital status, number of children, graduate, type of housing, work activity, social organization to which the participant belongs, activities in their free time; economic factors such as: work status, average monthly economic income, service time, main material of housing, ownership of housing, basic services; and, cultural factors: language skills, TV preferences, media information, parents' degree of education, place of birth, place of residence, social networks.

Table 10 shows the Wald score for the tested model, which indicates that the independent variables contribute significantly to the prediction of the dependent variable, the results obtained can be generalized to the population (Wald = 21.179 and p-value = 0.000 for employment status, Wald = 4.567 and p-value = 0.033 for basic electricity service, Wald = 6.136 and p-value = 0.013 for the preference for movies, Wald = 4.962 and p-value = 0.026 for media communications: TV and radio). Being the equation of the logit model the following:

Eq. (A.2) $$\begin{array}{l} \mathrm{elog}\;it=e-7.141+0.8055\;cond.lab\;\left(1\right)+4.502\;cond.lab\;\left(2\right)+3.292\;electri+3.164\;peliculas-2.152TV\;y\;radio\\ \log\;it=-7.141+0.8055\;cond.lab\;\left(1\right)+4.502\;cond.lab\;\left(2\right)+3.292\;electri+3.164\;peliculas-2.152TV\;y\;radio\\ \mathrm{elog}\;it=e-7.141+0.8055\;empl.stat\;\left(1\right)+4.502\;empl.sta\left(2\right)+3.292\;electri+3.164\;movies-2.152\;TV\;and\;radio\\ \log\;it=-7.141+0.8055\;empl.stat\;\left(1\right)+4.502\;empl.sta\left(2\right)+3.292\;electri+3.164\;movies-2.152\;TV\;and\;radio \end{array}$$

**Table 10:** Logistic model.

Variables	B	Standard error	Wald	gl	NXT.	Exp(B)
Employment status			22.137	2	.000	
Employment status (1)	8.055	1.750	21.179	1	.000	3148.189
Employment status (2)	4.502	1.179	14.573	1	.000	90.230
Electricity (1)	3.292	1.540	4.567	1	.033	26.896
Movies (1)	3.164	1.277	6.136	1	.013	23.667
TV and radio (1)	-2.152	.966	4.962	1	.026	.116
Constant	-7.141	1.936	13.605	1	.000	.001

### 3.2 Logit model to determine the social factors that influence job placement

Table 11 shows the logistic model of the social factors that influence the labor insertion of graduates of the National University of Moquegua.

**Table 11:** Classification tables between the observed and predicted values of the social factors that influence the labor insertion of graduates of the National University of Moquegua.

Observed		Predicted		
		Employment status		Correct percentage
		Does not work	Work	
Employment status	Does not work	36	22	62.1
	work	27	36	57.1
Overall percentage				59.5

According to the logistic regression analysis shown in classification Table 11, it indicates that there is a 59.5% probability of success in the result of the dependent variable, when the independent variable is known.

The Wald score for the logistic model indicates that the independent variable contributes significantly to the prediction of the dependent variable, and consequently it is concluded that the results obtained can be generalized to the population (Wald = 4.409 and p-value = 0.036 for the sport activity) (see Table 12).

**Table 12:** Logistic regression model of the social factors that influence the labor insertion of graduates of the National University of Moquegua.

	B	Standard error	Wald	gl	NXT.	Exp(B)
Sports (1)	.780	.372	4.409	1	.036	2.182
Constant	-.288	.255	1.277	1	.258	.750

The equation of the logit model is as follows:

Eq. (A.3) $$\begin{array}{l} e^{log\;it}=e^{-0.288+0.780\;deportes}\\ log\;it=-0.288+0.780\;deportes\\ e^{log\;it}=e^{-0.288+0.780\;sports}\\ log\;it=-0.288+0.780\;sports \end{array}$$

It means that 0.780 is the expected change in the logit model when changing sports activity, assuming the rest of the variables in the model are stable. The e ˆ 0.870 = 2.182, indicates that, when changing sports activity, the labor insertion of graduates doubles, that is, the option of labor insertion in graduates compared to non-labor insertion is multiplied by 2.182.

### 3.3 Logit model to determine the economic factors that influence job placement

Table 13 shows the logistic model of the economic factors that influence the labor insertion of graduates of the National University of Moquegua.

**Table 13:** Classification tables between the observed and predicted values of the economic factors that influence the labor insertion of graduates of the National University of Moquegua.

Observed		Predicted		
		Employment status		Correct percentage
		Does not work	Work	
Employment status	Does not work	43	15	74.1
	Work	2	61	96.8
Porcentaje global				86.0

For the logistic regression analysis, the classification table indicates that there is an 86% probability of success in the result of the dependent variable, when the independent variable is known.

The Wald score for the tested model indicates that the independent variables contribute significantly to the prediction of the dependent variable, therefore, the results obtained can be generalized to the population (Wald = 27.870 and p-value = 0.000 for employment status (1), Wald = 19.067 and p-value = 0.000 for Employment status (2) and Wald = 4.177 and p-value = 0.041 for basic electricity service) (see Table 14).

**Table 14:** Logistic regression model of the economic factors that influence the labor insertion of graduates of the National University of Moquegua.

	B	Standard error	Wald	gl	NXT.	Exp(B)
Employment status			31.559	2	.000	
Employment status (1)	6.548	1.240	27.870	1	.000	697.754
Employment status (2)	3.537	.810	19.067	1	.000	34.380
Electricity (1)	2.664	1.303	4.177	1	.041	14.348
Movies (1)	-5.641	1.483	14.474	1	.000	.004

The equation of the logit model is as follows:

Eq. (A.4) $$\begin{array}{l} e^{log\;it}=e^{-5.641+6.548\;cond.\;lab\;\left(1\right)+3,537\;cond.\;lab\;\left(2\right)\;+2.664\;electricidad}\\ \log\;it=-5.641+6.548\;cond.\;lab\;\left(1\right)+3,537\;cond.\;lab\;\left(2\right)\;+2.664\;electricidad\\ e^{log\;it}=e^{-5.641+6.548\;empl.sta\;\left(1\right)+3,537\;empl.\;sta\;\left(2\right)\;+2.664\;electricity}\\ \log\;it=-5.641+6.548\;empl.sta\;\left(1\right)+3,537\;empl.\;sta\;\left(2\right)\;+2.664\;electricity \end{array}$$

It means that 2.664 is the expected change in the logit model when the electricity service improves, assuming the rest of the variables in the model are stable.

### 3.4 Logit model to determine the cultural factors that influence job placement

Table 15 shows the logistic model of the cultural factors that influence the labor insertion of graduates of the National University of Moquegua.

**Table 15:** Classification tables between the observed and predicted values of the cultural factors that influence the labor insertion of graduates of the National University of Moquegua.

Observed		Predicted		
		Employment Status		Correct percentage
		Does not work	Work	
Employment Status	Does not work	13	45	22.4
	Work	6	57	90.5
Overall percentage				57.9

For the logistic regression analysis, the classification table indicates that there is a 57.9% probability of success in the result of the dependent variable, when the independent variables are known.

The Wald score for the logistic model indicates that the independent variables contribute significantly to the prediction of the dependent variable, the results obtained can be generalized to the population (Wald = 6.468 and p-value = 0.009 for movies and Wald = 4.929 and p -value = 0.026 for information media on TV and radio) (see Table 16).

**Table 16:** Logistic regression model of the cultural factors that influence the labor insertion of graduates of the National University of Moquegua.

	B	Standard error	Wald	gl	NXT.	Exp(B)
Movies (1)	1.222	.468	6.827	1	.009	3.393
TV and radio (1)	-1.004	.452	4.929	1	.026	.366
Constant	.045	.238	.035	1	.851	1.046

Presentation of the equation of the logit model:

Eq. (A.5) $$\begin{array}{l} e^{log\;it}=e^{0.045+\;1.222\;peliculas-1.004\;TV\;y\;radio}\\ \log\;it=0.045+\;1.222\;peliculas-1.004\;TV\;y\;radio\\ e^{log\;it}=e^{0.045+\;1.222\;movies-1.004\;TV\;media\;and\;radio}\\ \log\;it=0.045+\;1.222\;movies-1.004\;TV\;and\;radio \end{array}$$

It means that 1.222 is the expected change in the logit model when changing preference in TV, assuming stable the rest of the variables in the model and 1.004 is the expected change in the logit model when changing from towards the media communications: TV and radio.

### 3.5 Test statistic

The test statistic used was Chi-square, the results of which are shown in Table 17.

**Table 17:** Omnibus tests of model coefficients.

	Chi squared	gl	Sig.
Step	101.807	5	.000
Block	101.807	5	.000
Model	101.807	5	.000

The statistical efficiency score indicates that there is a significant improvement in the prediction of the probability of occurrence of the categories of the dependent variable (Chi-square: 101.807; p-value = 0.000 < 0.05). That is, the model improves the prediction of the dependent variable. Likewise, the Nagelkerke R squared value indicates that the proposed model explains 7.59 of the variance of the dependent variable (see Table 18).

**Table 18:** Model summary.

Logarithm of likelihood -2	Cox and Snell's R squared	Nagelkerke's R square
65.728a	.569	.759

a. The estimate has ended at iteration number 7 because the parameter estimates have changed by less than .001.

Therefore, as p-value = 0.000 of the omnibus tests of model coefficients, then it follows that the logistic multivariate model allows the identification of socioeconomic and cultural factors (employment status, electricity service, preference for movies and the media of TV and radio) that significantly influence the employment of graduates of the National University of Moquegua. Furthermore, Nagelkerke's R squared indicates that the proposed model explains 7.59 of the variance of the dependent variable. Regarding the residence of the graduates of the UNAM, it was found that 16.5% live in a rural area, 12.4% in a marginal urban area and 71.1% reside in an urban area. On the other hand, it is observed that 77.7% of UNAM graduates do not belong to any social organization, 2.5% of graduates belong to unions in the region, 15.7% to an association and 4,1% of graduates belong to a club in the Moquegua region.

## 4 Discussion

The approaches we have taken to develop a multivariate model have been different in order to identify the socioeconomic and cultural factors that influence the labor insertion of UNAM graduates. The results in general show that 30.6% of the graduates affirm that there are no job offers, while 69.4% of the graduates consider that there are. 52.1% were working, while 47.9% were not. In relation to social factors, it is observed that 92.6% of graduates are single, 5% single and 2.5% partner, 71.1% have their homes located in urban areas, 16.5% in rural areas and 12.4% in marginal urban areas. 78% do not belong to any social association, while 16% do belong.

Regarding economic factors, 30.6% do not receive any type of income, 47.9% have a monthly income of less than S/. 1200 soles (aprox. USD $343.00) 71.1% reside in a home built of bricks, 10.7% of mat, 9.1% of adobe, 5% of wood and 4.1% of stone and clay. 78.5% live in a family home, 11.6% rented or own, 6.6% own due to invasion and 3.3% in their own home. Regarding cultural factors, 68.6% speak Spanish, 5% Quechua and Spanish, 19% Spanish and English, and 7.4% speak three languages (Spanish, English and another language). 64.5% are from Moquegua, 16.5% from Puno, 4.1% from Tacna, 5% from Arequipa and 9.9% are from other cities. 2.5% of the mothers of the graduates have no studies, 17.4% have a primary educational level, 40.5% a secondary educational level, 24% a technical level and 15.7% a higher educational level. 7.4% of the parents of the graduates have no studies, 19.8% have a primary educational level, 50.4% a secondary educational level, 12.4% a technical level and 9.9% an educational level higher.

One study aimed to evaluate the statistical probability that a set of predictive variables (university degree, gender and residence of the graduates, as well as their complementary training) exert on a collection of variables (criterion, current and past employment, time in find the first job, as well as the monthly salary of the graduates). For this purpose, a survey study was implemented using a mixed strategy (postal and telephone), whose data have been subjected to the calculation of the binomial logistic regression in four different models. From the results obtained, it can be affirmed that the variable degree has been revealed as the most determining factor in at least two of them (employment rate and placement). The rest of the predictive variables have not been decisive (Rodríguez Sabiote, 2007).

Another study carried out in Spain, aimed to examine the evolution of graduates compared to graduates of extinct study plans; From which a negative aspect emerges, linked to the employability of graduates in functions and job categories that have nothing to do with what was studied, being a third of the graduates of the degree (Barba Aragón et al., 2016).

A study in Spain aimed to observe whether university graduates from more disadvantaged social origins are more unemployed and more inactive than those from households with more resources, it was based on bivariate statistical techniques and multivariate analysis (binomial logistic regression). It shows that even taking into account the degree, a significant influence of social origin is not observed in the labor insertion of this specific cohort of university graduates (Fachelli and Navarro-Cendejas, 2015).

A Mexican study aimed to analyze three dimensions of the survey of graduates to account for what the transit of graduates is like once they finish their studies, until they get a job or not. It was a quantitative study of non-experimental design and descriptive scope, where 11,444 graduates only from the higher level participated. 44.5% reported not having a job at the time of application and 50.2% detailed that they did. The main difficulty in getting a job was not having a degree (30.2%), what influenced them to get it was the coincidence of training with the needs of the company (80%). Most of those who work are located in micro-enterprises. In general, the graduates of this university reported high percentages of job satisfaction, with salary and hierarchical position attained, the aspects where they report less job satisfaction (Zalapa Lúa et al., 2019).

Another study in Mexico aimed to analyze the differences in job placement and their relationship with the type of public and private institution, taking as indicators: the rate of incorporation, the degree of coincidence between academic training and employment, as well as working conditions. The database obtained from the PROFLEX survey was analyzed, it was carried out in nine institutions in Mexico: six state public and three elite private. The results show that graduates of private Higher Education Institutions apparently have better working conditions in terms of salary, job stability and positions held. However, they do not have a rapid job insertion compared to their colleagues who graduated from public schools (García and Ulloa, 2018).

Therefore, the results obtained through the application of logistic regression models shows that employment condition, basic electricity services, the preference for movies, and the media communications: TV and radio, significantly influence graduates labor insertion from the National University of Moquegua.

## 5 Conclusion

It is concluded that, based on multivariate logistic regression, socio-economic and cultural factors influence the labor insertion of graduates of the National University of Moquegua. Being sports activity (social factor), the graduate's employment status and basic electricity services (economic factors), the preference for movies and media communications: TV and radio (cultural factors) which significantly influence in the labor insertion of graduates of the National University of Moquegua.

An important fact is that 77.7% of UNAM graduates do not belong to any social organization, only 2.5% of graduates belong to unions in the region, 15.7% to an association and 4.1% of graduates belong to a club in Moquegua region.

## Declarations

### Author contribution statement

Pedro Jesús Maquera-Luque: Conceived and designed the experiments; Performed the experiments; Wrote the paper.

José Luis Morales-Rocha: Conceived and designed the experiments; Analyzed and interpreted the data; Contributed reagents, materials, analysis tools or data; Wrote the paper.

Cynthia Milagros Apaza-Panca: Analyzed and interpreted the data; Wrote the paper.

### Funding statement

The research was funded by the National University of Moquegua (Approved with Resolution of the Organizing Commission No. 567-2017-UNAM).

### Data availability statement

Data will be made available on request.

### Declaration of interests statement

The authors declare no conflict of interest.

### Additional information

No additional information is available for this paper.
